# Automated quantification of levels of breast terminal duct lobular (TDLU) involution using deep learning

**DOI:** 10.1038/s41523-021-00378-7

**Published:** 2022-01-19

**Authors:** Thomas de Bel, Geert Litjens, Joshua Ogony, Melody Stallings-Mann, Jodi M. Carter, Tracy Hilton, Derek C. Radisky, Robert A. Vierkant, Brendan Broderick, Tanya L. Hoskin, Stacey J. Winham, Marlene H. Frost, Daniel W. Visscher, Teresa Allers, Amy C. Degnim, Mark E. Sherman, Jeroen A. W. M. van der Laak

**Affiliations:** 1 Department of Pathology, Radboud University Medical Center, Nijmegen, The Netherlands; 2 Radboud University Medical Center, Radboud Institute for Health Sciences, Nijmegen, The Netherlands; 3 Quantitative Health Sciences, Mayo Clinic, Jacksonville, FL USA; 4 Department of Cancer Biology, Mayo Clinic, Jacksonville, FL USA; 5 Department of Laboratory Medicine and Pathology, Mayo Clinic, Rochester, MN USA; 6 Health Sciences Research, Rochester, MN USA; 7 Division of Medical Oncology, Mayo Clinic, Rochester, MN USA; 8 Department of Surgery, Mayo Clinic, Rochester, MN USA; 9 Center for Medical Image Science and Visualization, Linköping University, Linköping, Sweden

**Keywords:** Translational research, Predictive markers, Breast cancer

## Abstract

Convolutional neural networks (CNNs) offer the potential to generate comprehensive quantitative analysis of histologic features. Diagnostic reporting of benign breast disease (BBD) biopsies is usually limited to subjective assessment of the most severe lesion in a sample, while ignoring the vast majority of tissue features, including involution of background terminal duct lobular units (TDLUs), the structures from which breast cancers arise. Studies indicate that increased levels of age-related TDLU involution in BBD biopsies predict lower breast cancer risk, and therefore its assessment may have potential value in risk assessment and management. However, assessment of TDLU involution is time-consuming and difficult to standardize and quantitate. Accordingly, we developed a CNN to enable automated quantitative measurement of TDLU involution and tested its performance in 174 specimens selected from the pathology archives at Mayo Clinic, Rochester, MN. The CNN was trained and tested on a subset of 33 biopsies, delineating important tissue types. Nine quantitative features were extracted from delineated TDLU regions. Our CNN reached an overall dice-score of 0.871 (±0.049) for tissue classes versus reference standard annotation. Consensus of four reviewers scoring 705 images for TDLU involution demonstrated substantial agreement with the CNN method (unweighted κappa = 0.747 ± 0.01). Quantitative involution measures showed anticipated associations with BBD histology, breast cancer risk, breast density, menopausal status, and breast cancer risk prediction scores (*p* < 0.05). Our work demonstrates the potential to improve risk prediction for women with BBD biopsies by applying CNN approaches to generate automated quantitative evaluation of TDLU involution.

## Introduction

Deep learning pathology methods have demonstrated potential utility in critical diagnostic applications, including prostate cancer grading^1,2^, and detection of lymph node metastases in breast cancer (BC)^3^. These methods are particularly suited to quantify multiple morphologic features, which could transform pathology diagnosis from a largely qualitative and subjective discipline, to one that incorporates objective measurements that cannot be accomplished routinely by visual assessment. The assessment of involution of terminal duct lobular units (TDLUs), which represent the structures from which early BC precursors arise (i.e. benign breast disease or BBD) provides a notable example. TDLUs are the functional units of the breast that produce milk after childbirth and represent the source of most BC precursors^4,5^. TDLUs are composed of epithelial sub-structures termed acini and terminal ducts embedded in stroma containing immune cells and vessels. TDLU involution, a gradual physiologic process that often begins in the fourth decade of life, results in simplification of lobules (reduction in acini size and number) and decreased TDLU density (reduction in lobule span and lobules per unit area)^6^.

Increased levels of TDLU involution in BBD biopsies, as assessed visually, have been related to lower BC risk in large cohorts; however, the lack of automated and quantitative methods for assessing this feature poses barriers to its potential clinical implementation^6–8^.

Data indicate that delayed involution (i.e. greater preservation of TDLU numbers and structure with aging) modifies BC risk among women with BBD, and complements other risk factors^6–9^. Recently, first steps in automated assessment of age-related TDLU involution have been made, in which deep learning was used for detection of acini, and segmentation of lobular area and adipose tissue^10^. Although this method generally agreed with manually defined annotations, it did not predict BC risk among patients with BBD, as has been demonstrated by subjective and morphometric analyses of TDLU involution in prior reports^6–9,11,12^. Thus, further studies are needed both to assess technical performance of deep learning methods and to assess the relationship of TDLU involution and breast cancer risk.

Accordingly, we developed convolutional neural networks (CNNs) to measure involution in background TDLUs included in BBD biopsies and to preliminarily demonstrate their relationships with important clinical factors. We describe the development and preliminary validation of a quantitative approach using CNNs to automatically segment and characterize individual TDLUs.

## Results

### Performance of the CNN tissue segmentation

**Fig. 1:**
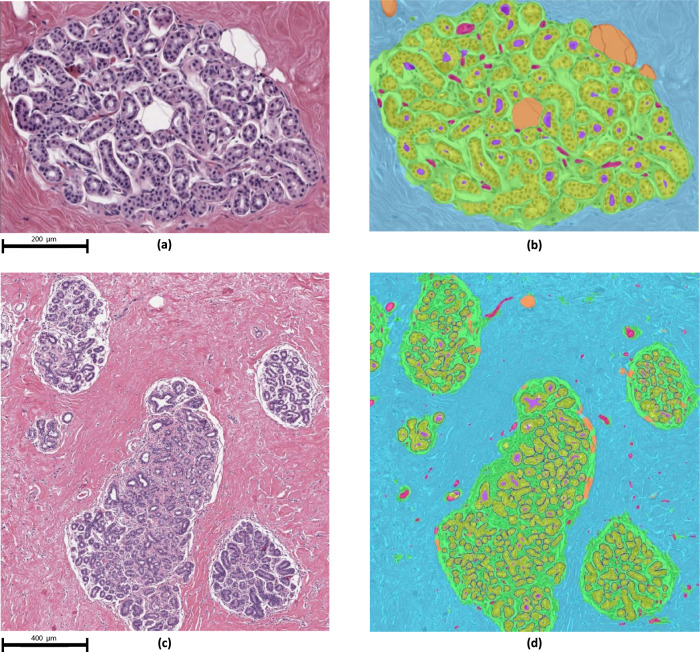
Structure segmentation visual results: Visual examples of the segmentation results. The top view (**a**, **b**) shows a single TDLU with epithelium borders removed, with (**a**) and without (**b**) segmentation overlay. The bottom view gives a broad overview (**c**, **d**). The classes are mapped as follows: epithelium (yellow), intralobular stroma (green), extralobular stroma (blue), lumen (purple), adipose tissue (orange), small vessel (pink), border (dark blue).

**Fig. 2:**
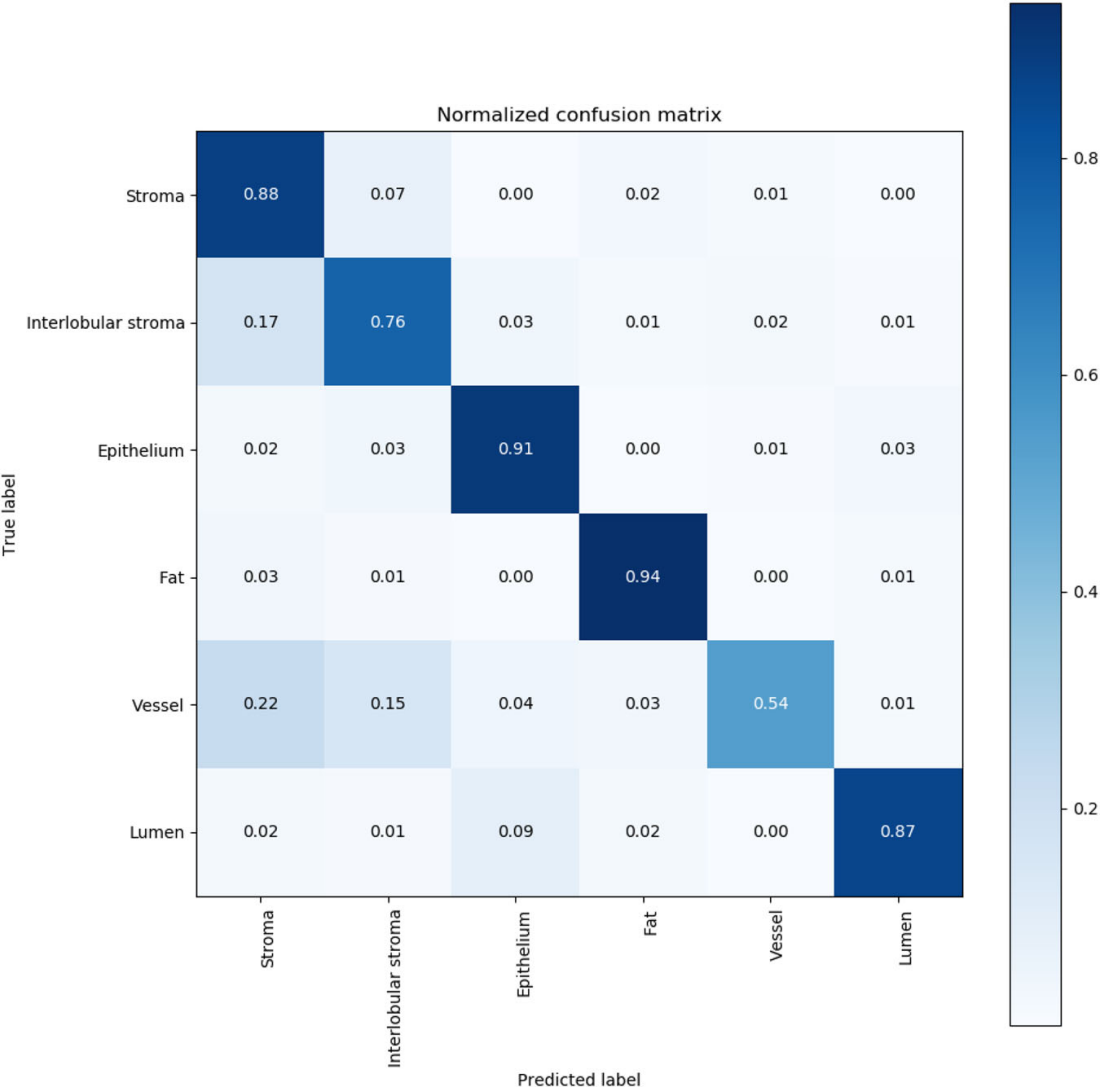
Confusion matrix of the structure segmentation results: confusion matrix of the segmentation results on the held-out test set. These matrices show how different classes may be misclassified. The vertical axis displays the ground truth label and the horizontal axis shows the label that was predicted by the neural network. As an example: for the lumen class 85% of the pixels are correctly classified and 10% of the pixels are misclassified as epithelium.

Our CNN was trained and independently tested on 13 and 20 slides, respectively. Dice-scores for the segmentation among the six structure classes were lowest for capillaries (0.568) compared to other features (weighted mean 0.871; range = 0.768–0.942) (Table 1). Representative examples of the segmentation of TDLU areas are shown in Fig. 1, which visually confirms the accuracy of the CNN in identifying specific tissue components within TDLUs. Although there were few discordances, areas of misclassification between the CNN and ground truth visual classifications included confusion between extralobular stroma versus intralobular stroma and vessels versus stroma of both types (Fig. 2).

**Table 1:** Structure segmentation Dice-scores: Dice-scores of the structure classes on the held-out test set of 20 slides.

Structure Class	Dice-score
Extralobular stroma	0.882 (±0.032)
Intralobular stroma	0.768 (±0.060)
Epithelium	0.917 (±0.019)
Fat	0.942 (±0.011)
Vessel	0.568 (±0.214)
Lumen	0.839 (±0.103)
All classes (weighted mean)	0.871 (±0.049)

The Dice-score is a measure of agreement between the segmentation output by the CNN and the annotated ground truth. Confidence intervals were obtained using bootstrapping.

### Inter-rater agreement in classification of TDLU involution scores

Four independent reviewers individually assessed up to ten TDLUs in 161 slides, according to predefined criteria (Supplementary Table 1). Of the 705 TDLUs scored, 572 (81%) TDLUs were included in our analysis, after excluding images that were rated as unsatisfactory quality by at least one reader (See the quality section on the data collection form, Supplementary Table 1). Substantial levels of agreement were reached among all individual readers (Table 2), ranging from kappa = 0.656 (95% CI: 0.655–0.657) between reader 1 vs. 4, to kappa = 0.748 (95% CI: 0.747–0.749) between reader 3 vs. 4. The consensus counts for each of the six TDLU involution levels were: 0: 90, 1: 46, 2: 51, 3: 68, 4: 166, 5: 151. Supplementary Table 2 shows the individual agreements among the readers. Supplementary Figure 1 shows the correlation between the original *none, partial, complete* labels (Table 3), which have been correlated with risk among over 13,000 patients^13^, and the consensus of the 6 levels of involution as scored by the readers.

**Table 2:** Reader study inter-observer agreement: Kappa statistics for agreement in level of TDLU involution between four individual readers, the consensus of their reads, and the CNN.

Anonymized reader	Cohen’s Kappa score (95% Confidence interval)
Reader 1	0.687 (0.686–0.688)
Reader 2	0.662 (0.661–0.663)
Reader 3	0.729 (0.728–0.731)
Reader 4	0.680 (0.679–0.681)
Consensus	0.747 (0.746–0.748)

Confidence intervals were obtained using bootstrapping.

**Table 3:** Patient Characteristics: Characteristics of patients included in analysis set for this study.

	BBD case (*N* = 87)	BBD control (*N* = 87)	Total (*N* = 174)
Age			
Median	52	52	52
Range	(35–74)	(35–74)	(35–74)
Age category, *n* (%)			
<45 years	19 (21.8%)	19 (21.8%)	38 (21.8%)
45–55 years	33 (37.9%)	33 (37.9%)	66 (37.9%)
>55 years	35 (40.2%)	35 (40.2%)	70 (40.2%)
BBD histology *n* (%)			
Non-proliferative	26 (29.9%)	40 (46.0%)	66 (37.9%)
Proliferative disease without atypia	42 (48.3%)	37 (42.5%)	79 (45.4%)
Atypical hyperplasia	19 (21.8%)	10 (11.5%)	29 (16.7%)
Age-related lobular involution, *n* (%)			
Involution data missing	7	2	9
None	22 (27.5%)	15 (17.6%)	37 (22.4%)
Partial	44 (55.0%)	39 (45.9%)	83 (50.3%)
Complete	14 (17.5%)	31 (36.5%)	45 (27.3%)

### Agreement between automated method and visually scored levels of TDLU involution

Substantial levels of agreement between individual readers and automated method ranged from kappa = 0.662 (95% CI: 0.661–0.663) to 0.729 (95% CI: 0.728–0.731) with agreement of the consensus visual read versus CNN yielding a kappa of 0.747 (95% CI:0.746–0.748) (Table 2).

### Distributions of quantitative features

Our automatically extracted quantified features on TDLU tissue regions revealed strong positive associations between acini count, epithelial size, and TDLU size; and between capillary count and capillary size. Moderate associations were seen for most other paired sets of features. The epithelial-to-stromal ratio was not strongly correlated with any of the other features. A scatterplot matrix of the individual features is shown in Supplementary Figure 2.

### Comparison quantitative features and clinical data

**Fig. 3:**
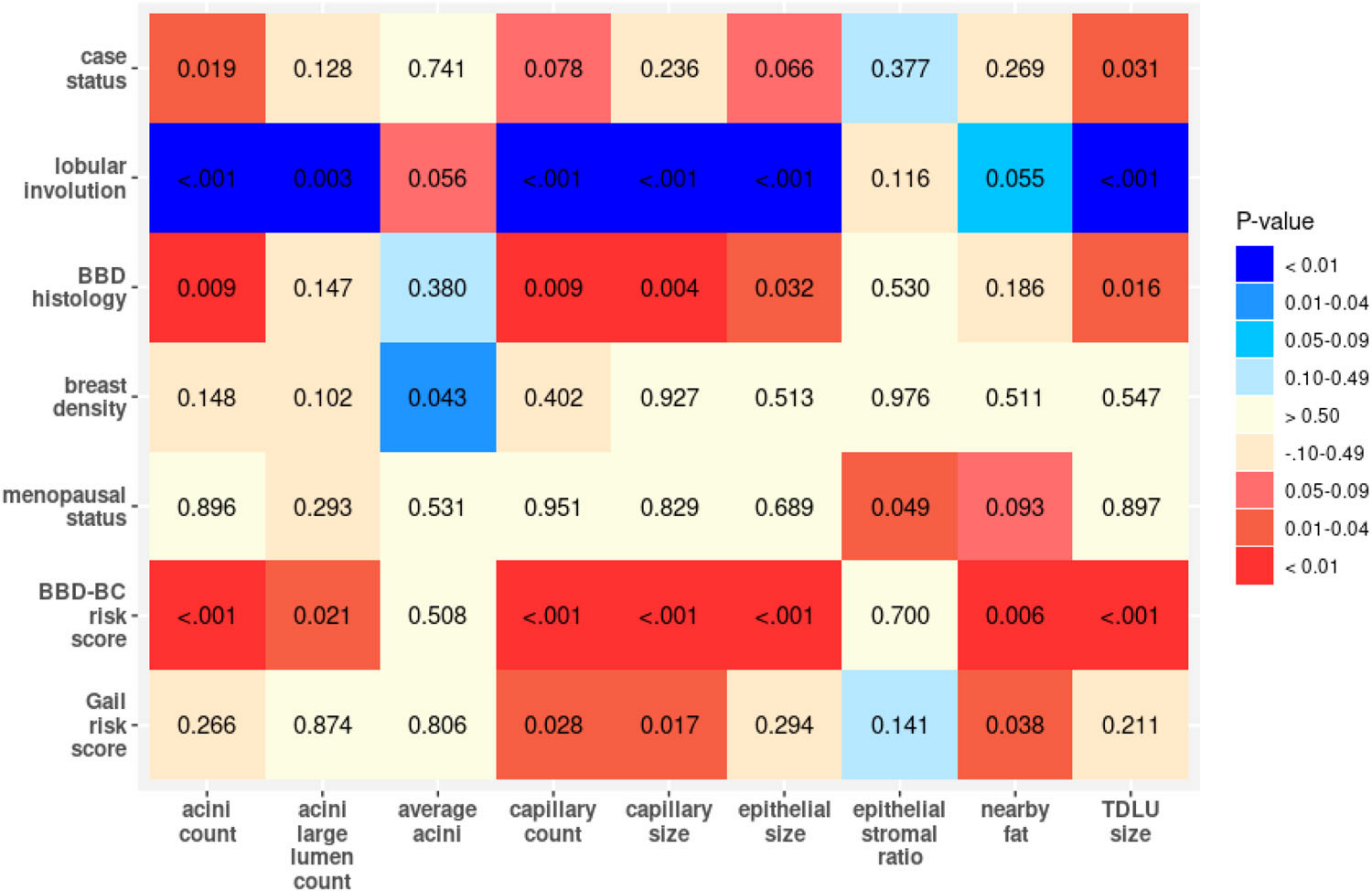
Analysis of AI-derived features versus clinical variables: age-adjusted associations of AI-derived breast biopsy biomarkers with demographic and clinical variables of subjects. Numbers indicate *p* values of association. Blue shades indicate negative associations between demographic/clinical variable and AI feature (i.e., feature value decreases as variable increases), whereas red shades indicate positive associations.

Figure 3 reports the *p* values of the age-adjusted results for individual quantified features per subject and the clinical measurements; reported *p* values are not corrected for multiple testing given the exploratory nature of analyses. Nearly all AI measures were significantly associated with the previously performed subjective evaluations of TDLU involution. Other AI measures of involution showed association to varying degrees with clinical features such as severity of BBD, BC risk by the BBD-BC or Gail models, and case versus control status. AI measures showed only a single marginally significant association with breast density. Detailed association results of AI-derived features with demographic and clinical variables are shown in Supplementary Tables 3 and 4.

## Discussion

Increased levels of TDLU involution in BBD biopsies, as assessed visually or morphometrically, have been associated with lower BC risk^6–8,13^. However, lack of standardized criteria for assessing involution and the effort required for its assessment have slowed research on this topic and impeded prospects for its translation into clinical practice. Increasing use of digital slide images for research and diagnosis creates an unprecedented opportunity to develop and apply automated computational approaches in pathology, including assessment of TDLU involution in BBD biopsies. Accordingly, we developed an automated method for assessment of TDLU involution in BBD biopsies, using CNNs. Our CNN demonstrated: (1) accurate segmentation of relevant breast tissue structures; (2) agreement with TDLU involution levels based on visual consensus comparable to that found among different raters and (3) expected associations with multiple clinical and pathologic features. Similar to many pathology classifications, current BBD classification reflects only the most high-risk finding in a specimen, while ignoring the totality of changes present throughout the tissue. Therefore, computational pathology approaches such as we describe, which comprehensively and objectively quantify histologic features, offer the potential to transform pathology diagnosis^14^.

Classic papers by Henson and Tarone suggested that involution of TDLUs may be related to BC risk and to mammographic density, an important BC risk factor that reflects the proportion of fibroglandular tissue in the breast^15^. Further, these authors related the initiation of TDLU involution in the fourth decade of life to the slower rise in BC age-specific incidence as women approach menopause and suggested that assessment of involution in benign biopsies might have important utility in predicting BC risk^16^. This view is supported by studies showing that TDLU involution is downstream of established breast cancer risk factors, including elevated serum levels of estradiol, testosterone, prolactin, and growth factors, and therefore, delayed age-related TDLU involution may represent an intermediate state in breast carcinogenesis^17,18^.

Initial attempts to assess levels of involution in human samples were based on subjective impressions of whole slides, wherein TDLU involution classified as none, partial or complete predicted BC risk in a large BBD cohort^7^. While this approach was imprecise and subjective, it enabled rapid human analysis of thousands of biopsies and assessment of risk^19^. Nonetheless, the lack of an automated, standardized approach for scoring TDLU involution has posed a barrier to expanding research and clinical translation. Early work on quantitative measures, such as TDLU density, acini count per TDLU and TDLU span, confirmed associations between TDLU involution and BC risk, but these measurements were not automated, and therefore, labor intensive^6,9,20^.

In this report, we established a CNN to automatically segment relevant tissue structures throughout a whole slide image. Dice-scores indicate that our segmentation performed particularly accurately with regard to classification of epithelium, fat, and lumen, with dice-scores of 0.85-0.90. Disagreement between “reference standard” expert annotation and the automated segmentation was found predominantly for classifying stroma and capillaries. The boundary between extralobular and intralobular stroma frequently becomes ill-defined as women age and specialized intralobular stroma are replaced by denser fibrous stroma, which merges with extralobular stroma. Similarly, visual annotation of small vessels (presumptive capillaries) without immunostaining for endothelial markers may be challenging for both humans and computers. Given evidence linking vascularity to markers of BC risk, such as benign parenchymal enhancement as assessed by magnetic resonance imaging, and the association of increased microvessel density with BC risk, developing improved recognition of microvessels may have value for risk assessment and research integrating pathology and radiologic imaging^21^. Our segmentation network effectively characterized TDLU involution, yielding a Kappa = 0.74 (Table 3) versus the consensus of four reviewers, applying a six-level scale, signifying substantial agreement. Assessment of TDLU involution in routinely prepared histopathologic 5-um tissue sections cannot fully capture features of a three-dimensional process. However, this applies equally to all samples, and this is unbiased misclassification that would not substantially alter our interpretations.

In addition to expected associations with independently assigned subjective involution status, significant expected relationships were found for severity of BBD classification, breast density, menopausal status, and scores of BC risk prediction models. AI features showed greater associations with the BBD-BC risk prediction model, which incorporates TDLU involution, than for the Gail model which does not include this parameter. Further, relationships between involution and mammographic density were limited, consistent with prior finding that these two factors are independent predictors of risk among women with BBD^22^. Our analyses suggest the potential relevance of AI features beyond TDLU involution, justifying further research into using AI for automated assessment.

Recently, Kensler et al applied a CNN method to BBD biopsies to assess BC risk in a nested case–control study^12^. Although visually assessed TDLU involution was previously reported to be associated with BC risk in this cohort (Baer et al.), the CNN results were not related to BC risk, prompting the authors to conclude that TDLU involution was not a strong BC risk factor in their cohort^8^. However, a potential concern with their AI analysis is the absence of expected reductions in TDLU span, TDLU counts/unit area, or median / mean acini counts per TDLU with increasing age among their premenopausal controls. TDLU involution likely begins well before menopause in many women, and has been characterized robustly in prior studies. Analysis of 1,938 benign breast tissues donated for research showed declines in TDLU counts (RR-0.87, 95% CI = 0.83–0.91) and acini counts / TDLU (RR = 0.54, 95%CI = 0.40–0.74) at ages 40–49 years versus women aged <40 years, and declines in TDLU span between ages 30–39 years (OR = 0.64, 95% CI = 0.48–0.87) versus even younger women^6^. Findings from the Mayo BBD cohort also demonstrate that involution is underway by the 3rd decade of life, with some degree of involution observed in 45.6% of women aged <30 years and in 73.7% of women aged 40–49 years^7^. Furthermore, in updated analyses of 13,485 cohort participants, visual assessment of TDLU involution, masked to follow-up data, remained significantly predictive of BC risk^13^. Thus, TDLU involution may be a feature among BBD patients aged <50 years who remain BC-free and distinguishes them from those who later develop BC. As development of computational methods is in their infancy, we believe it is premature to conclude that TDLU involution is not associated with BC risk, and we remain optimistic that automated CNN approaches will improve assessment of BC risk in BBD biopsies.

A limitation in our study was the modest sample size; nonetheless, our analyses generated precise estimates of associations with substantial statistical significance. Furthermore, the CNN was trained using extensive image augmentations to make it robust to unseen stain variations^23^. These findings support proceeding with larger confirmatory studies.

In summary, our data show that a CNN can provide an automated approach for quantifying TDLU involution, a potential marker of BC risk in BBD biopsies. Potential future research may include development of deriving a continuous score for age-related lobular involution. This would allow for continuous assessment of involution versus age, which may be important given that lack of progressive involution in sequential BBD biopsies predicts increased BC risk^11^. Although our study is limited to measuring TDLU involution, it provides a necessary first step towards quantitatively characterizing BBD histopathology and demonstrates the potential convergence of pathology, computer science and epidemiological risk assessment. In future work, we aim to develop a fully automated system to analyze BBD biopsies that will evaluate all lobular structures, define normal and BBD lobules, and provide quantitative metrics for each lobule, including level of TDLU involution for normal lobules.

## Methods

### Tissue samples

This analysis evaluated BBD biopsies from an existing case–control set of 174 patients selected from the pathology archives at Mayo Clinic, Rochester, MN between 1992 and 2001^19,24^ (Table 3). Patients were selected using a nested case–control design to include 87 cases who developed BC during follow-up after BBD biopsy and 87 controls who were BC-free with follow-up duration greater than the longest time-to-cancer among the cases; controls were additionally frequency matched to the age distribution of the BBD cases in the categories of age <45, 45–55, and >55. Median age of women at biopsy was 52 years (range 35–74 years), including 66 (37.9%) with non-proliferative BBD, 79 (45.4%) with proliferative BBD without atypia, and 29 (16.7%) with atypical hyperplasia. Of the 29 cases with atypical hyperplasia, 14 had ductal hyperplasia, 14 had lobular hyperplasia and 1 case had both ductal and lobular hyperplasia. This sample set was chosen because it is representative of BBD biopsies from our BBD cohort and had been rigorously reviewed and was annotated with epidemiologic data.

**Fig. 4:**
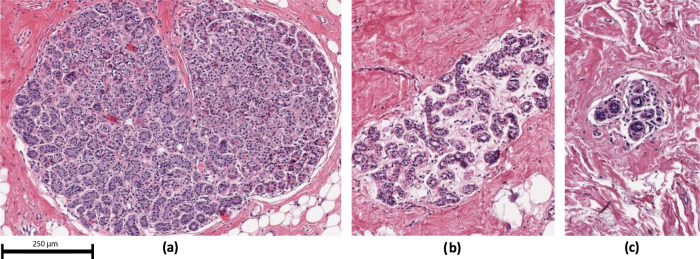
Involution examples: Examples of different levels of involution. From left to right: no involution (**a**), partial involution (**b**), complete involution (**c**).

Routinely prepared hematoxylin and eosin-stained sections from the clinical archive at the Mayo Clinic were reviewed previously by an expert pathologist (DV) masked to clinical outcome using a standardized data collection instrument to characterize the full spectrum of BBD changes and to qualitatively score the level of TDLU involution on an ordinal scale as none, partial or complete^7^. Examples of the three involution levels are shown in Fig. 4. All microscopic slides were digitized using the scanner with a 20x objective at a resolution of 0.495 μm per pixel. The project was approved by the Mayo Clinic Institutional Review Board.

We included 33 slides for training and testing our CNN system. An independent sample of 161 slides was included in our reader study to compare performance of the CNN with trained readers. There was no overlap between slides used in the training set of the CNN and slides used for the reader study.

### Automated method development and design

**Fig. 5:**
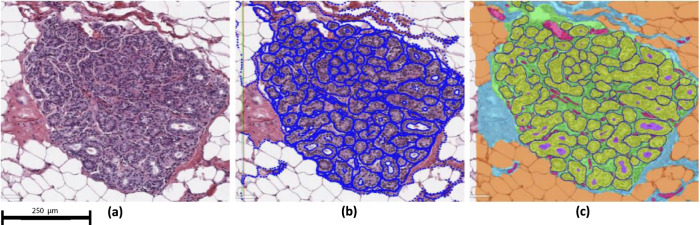
Annotation process example: An example demonstrating the process of annotation. A single TDLU region (**a**) is selected to be fully annotated (**b**). The annotations are then converted to a ground truth map (**c**) using ASAP. The classes are mapped as follows: epithelium (yellow), intralobular stroma (green), extralobular stroma (blue), lumen (purple), adipose tissue (orange), vessel (pink). The border class (dark blue) was separately added in a post-processing step.

To obtain a ground truth (i.e. “gold standard” classification) regarding tissue type/segmentation, a human observer randomly selected up to five TDLUs to be annotated in each of the selected training and evaluation slides. A rectangular region was placed around the marked TDLUs and subsequently exhaustively annotated, using the slide viewer ASAP (open source available at https://github.com/computationalpathologygroup/ASAP, version 1.9). Six relevant tissue types or features were identified: epithelium (and myoepithelium), extralobular stroma, intralobular stroma, adipose tissue, lumens of acini, and small caliber blood vessels (“capillaries”). The distinction between extralobular and intralobular stroma is defined by the proximity of epithelium, i.e., intralobular stroma lies between and around acini of the TDLUs. Vessel annotation was restricted to small caliber vessels, as approximating the caliber of capillaries at 25 μm, within and immediately surrounding the TDLUs. To facilitate the detection of individual acini, a seventh class was added to represent the border between neighboring objects. The epithelial border class was created by carefully annotating the perimeter of individual acini and converting the annotation outline to a border measuring on average 3 pixels (1.5 μm). An example annotation is shown in Fig. 5. An overall number of 3,027 annotations across 48 TDLUs from 13 slides were utilized to develop the CNN. We selected TDLUs distributed among the three involution classes: 12 TDLUs with no involution, 18 partially involuted, and 18 with complete involution. Due to the homogeneous appearance of the tissue classes, few annotations were required for good performance of our CNN. We tested our network on 88 annotated TDLUs, across the 20 remaining slides.

A CNN was trained for the delineation/segmentation task, applying the U-net architecture which has been proven to work well specifically for medical tissue segmentation^25^. Apart from image flipping and rotation, the neural network was trained with gaussian noise, gaussian blurring, and color augmentations, to account for potential variations induced during scanning and staining. Nine quantitative features, that are potentially linked to TDLU involution and may be used to discriminate between involution levels, were defined and extracted from each segmented TDLU: TDLU area, acini count, epithelial area, proportion of epithelial area vs. intralobular stroma area, small vessel count, small vessel area, adipose tissue area, average acinar size and acini with large lumen count. For the purpose of counting the acini, we merged the border class with the intralobular stroma class and counted all epithelial components with a total area larger than 800 pixels as a single unit. The total lobular area, defined above as TDLU area, is composed of the total epithelial, luminal, and intralobular stroma areas. Further details on the algorithms of the CNN and extraction of the quantitative features are elaborated in Supplementary Note 1.

### Reader study design

For visual assessment of individual TDLUs, we evaluated up to 10 previously annotated, representative “normal” appearing TDLUs showing varying levels of involution (but not features of BBD) per whole slide image (excluding CNN training slides, Supplementary Table 5) of each BBD biopsy (*n* = 161). Digital images for TDLU scoring were made available through the online viewer on the Grand Challenge website (https://grand-challenge.org) and linked to an electronic data collection form that included queries about adequacy for evaluation and levels of TDLU involution (Supplementary Table 1). Four independent reviewers, masked to all data, assessed levels of involution according to predefined criteria, defined based on features identified in prior analyses^6–9^. Specifically, features, and particularly categories of acini counts per TDLU, were developed based on distributions found in normal TDLUs within BBD biopsies^7^. Previously published studies demonstrate excellent intra- and inter-observer agreement among reviewers in assessing TDLU involution. Prior to the current review, readers participated in discussions of criteria for rating involution and training that included microscopic review^26^. Four readers (MES, MS, TH, JO) independently scored levels of involution in 705 normal TDLUs on a scale from 0–5 as follows: 0: >40 acini; 1 or 2: 26–39 acini; 3 or 4: 10–25 acini and 5: <10 acini. Distinctions of 1 versus 2 and 3 versus 4 were based on acini number plus qualitative features, with tighter packing of acini, less dense intervening stroma, and absence of basement membrane thickening favoring the relatively less involuted categories. This approach enabled reviewers to subjectively assess levels of TDLU involution when acini counts per TDLU were on the border between categories and to compensate for incomplete representation of TDLUs, which are three-dimensional structures that are evaluated in 5-μm sections. The most highly involuted TDLUs (category 5), were composed of small acini, often associated with basement thickening and densely hyalinized relatively avascular stroma lacking edema. Clearly defined acini were counted, irrespective of whether the lumen was identifiable in the plane of section. Any images judged ungradable by one or more reviewers were excluded. Reasons for exclusion were: uncertain identification of TDLU (i.e. possible ducts, without acini or interlobular ducts), presence of multiple TDLUs or poor quality due to histological artifacts. The full data collection form for each image is included in Supplementary Table 1.

### Comparison quantitative features with readers’ scores

A random forest model was developed to determine the TDLU involution score based on the nine automated measures extracted from the segmentation. The random forest was initialized with 500 trees, using the standard parameters of the scikit-learn package in Python. We used a tenfold stratified cross-validation approach to assess the model performance, which was compared to the consensus data from the four readers (majority vote, see details below). For each fold, 90% of the readers’ consensus data (defined below) was used to fit the model, and 10% of the data was used to calculate the performance of the model.

### Statistical analysis

Data were summarized using means and standard deviations for continuous variables, and tables and percentages for categorical variables. Performance of the CNN tissue segmentation, versus the ground truth tissue annotation based on visual review, was assessed with the Dice-score per individual structure class. The Dice-score, which is a measure of overlap between the segmentation output by the CNN and the annotated ground truth, is calculated as 2(|S | ∩ | G | )/( | S | + | G | ), where S is the segmentation map and G is the ground truth. The score can range from 0 (no agreement between assigned classes) and 1 (complete agreement). The overall Dice-Score is reported, which is calculated using the weighted (according to samples per class) mean across all structures. The multiclass segmentation performance was assessed on 20 held-out whole slide images.

Inter-rater agreement between the four readers was determined using the linear Cohen’s kappa statistic. Agreement scores are characterized with values <0 indicating no agreement, 0–0.20 slight, 0.21–0.40 fair, 0.41–0.60 moderate, 0.61–0.80 substantial, and 0.81–1 as almost perfect agreement^27^. We compared each individual reader with the majority vote of the other three readers. The rounded-up average was taken in case of a three-way split in scores. We calculated the consensus by using the majority vote of the four readers. For comparison with our automated method, we used the majority vote of all four readers. If no majority could be determined (in case of a two-way split or a four-way split), the average score (rounded up) was taken. Confidence intervals for the inter-rater agreements and Dice-scores were calculated by performing bootstrapping (*n* = 2,000) on the results^28^.

We used the mean quantitative feature across the included TDLUs per subject to compare with the demographic/clinical attributes. Prior to statistical comparisons, each CNN-derived quantitative feature was transformed using inverse normal (van der Waerden) scores to account for data skewness. The resulting scores can be interpreted much like z-scores from a standard normal (Gaussian) distribution. Pairwise associations of the CNN scores were examined using scatterplot matrices. In exploratory analyses, we compared the quantitative features with demographic and clinical variables using linear regression analyses. These analyses were performed to preliminarily assess whether AI measurements generally recapitulated associations observed with visual assessment and morphometry; the study was not designed and powered to test the performance of the AI system in predicting case–control status, which would require a larger dataset. For each pair of CNN score and demographic/clinical attribute, we examined unadjusted and age-adjusted associations. The following attributes were examined: BC case status (case, control); TDLU involution (none, partial, complete); BBD histology (non-proliferative disease [NP], proliferative disease without atypia [PDWA], atypical hyperplasia [AH]); breast density (low, high); ever parous (yes, no); menopausal status (pre-menopausal, post-menopausal); hormone replacement therapy (HRT) use (never, former, current); estrogen receptor (ER) status for the breast cancer cases (negative, positive); the BBD-BC 5-year breast cancer risk score (numeric value between 0 and 1); and the Gail Model 5-year breast cancer risk score (numeric value between 0 and 1)^29–31^. Resulting *p* values were displayed in matrix form in the style of a heat map. All statistical tests were two-sided. Due to the exploratory nature of the associations, *p* values < 0.05 were considered statistically significant.

### Reporting summary

Further information on research design is available in the Nature Research Reporting Summary linked to this article.

## Supplementary information

Reporting Summary

Supplementary Information

## Data Availability

The dataset supporting the conclusions of this article is upon request with adherence to HIPPA laws and the institutions’ IRB policies. For data requests please contact the corresponding (first) author.
