# Supplementary material for: Automated quantification of levels of breast terminal duct lobular (TDLU) involution using deep learning
Source: NPJ Breast Cancer. 2022 Jan 19;8:13. doi: 10.1038/s41523-021-00378-7 (PMC8770616; doi:10.1038/s41523-021-00378-7)
Supplement: Supplementary file 1 — Reporting Summary [file 41523_2021_378_MOESM1_ESM.pdf]

nature portfolio

Corresponding author(s): Thomas de Bel

Last updated by author(s): Dec 1, 2021

Reporting Summary

Nature Portfolio wishes to improve the reproducibility of the work that we publish. This form provides structure for consistency and transparency in reporting. For further information on Nature Portfolio policies, see our Editorial Policies and the Editorial Policy Checklist.

Statistics

For all statistical analyses, confirm that the following items are present in the figure legend, table legend, main text, or Methods section.

n/a	Confirmed	
☐	☒	The exact sample size (*n*) for each experimental group/condition, given as a discrete number and unit of measurement
☐	☒	A statement on whether measurements were taken from distinct samples or whether the same sample was measured repeatedly
☐	☒	The statistical test(s) used AND whether they are one- or two-sided<br>*Only common tests should be described solely by name; describe more complex techniques in the Methods section.*
☐	☒	A description of all covariates tested
☐	☒	A description of any assumptions or corrections, such as tests of normality and adjustment for multiple comparisons
☐	☒	A full description of the statistical parameters including central tendency (e.g. means) or other basic estimates (e.g. regression coefficient) AND variation (e.g. standard deviation) or associated estimates of uncertainty (e.g. confidence intervals)
☐	☒	For null hypothesis testing, the test statistic (e.g. *F*, *t*, *r*) with confidence intervals, effect sizes, degrees of freedom and *P* value noted<br>*Give P values as exact values whenever suitable.*
☒	☐	For Bayesian analysis, information on the choice of priors and Markov chain Monte Carlo settings
☒	☐	For hierarchical and complex designs, identification of the appropriate level for tests and full reporting of outcomes
☒	☐	Estimates of effect sizes (e.g. Cohen's *d*, Pearson's *r*), indicating how they were calculated

Our web collection on statistics for biologists contains articles on many of the points above.

Software and code

Policy information about availability of computer code

Data collection	No software was used for data collection
Data analysis	Analyses were carried out using Python 3.6; with the packages scikit-learn (0.20.3), NumPy (1.16.2), pandas (0.24.2), SciPy (1.2.1); and SAS Studio Version 3.8 (SAS Institute, Inc., Cary, NC).

For manuscripts utilizing custom algorithms or software that are central to the research but not yet described in published literature, software must be made available to editors and reviewers. We strongly encourage code deposition in a community repository (e.g. GitHub). See the Nature Portfolio guidelines for submitting code & software for further information.

Data

Policy information about availability of data

All manuscripts must include a data availability statement. This statement should provide the following information, where applicable:

- Accession codes, unique identifiers, or web links for publicly available datasets
- A description of any restrictions on data availability
- For clinical datasets or third party data, please ensure that the statement adheres to our policy

The dataset supporting the conclusions of this article is upon request with adherence to HIPPA laws and the institutions' IRB policies.

Field-specific reporting

Please select the one below that is the best fit for your research. If you are not sure, read the appropriate sections before making your selection.

☒ Life sciences ☐ Behavioural & social sciences ☐ Ecological, evolutionary & environmental sciences

For a reference copy of the document with all sections, see nature.com/documents/nr-reporting-summary-flat.pdf

Life sciences study design

All studies must disclose on these points even when the disclosure is negative.

Sample size	This analysis evaluated BBD biopsies from an existing case-control set of 174 patients selected from the pathology archives at Mayo Clinic, Rochester, MN between 1992 and 2001. Patients were selected using a nested case-control design to include 87 cases who developed BC during follow-up after BBD biopsy and 87 controls who were BC-free with follow-up duration greater than the longest time-to-cancer among the cases. This sample set was chosen because it is representative of BBD biopsies from our BBD cohort and had been rigorously reviewed and was annotated with epidemiologic data.
Data exclusions	13 slides were excluded from the reader study, because they were used for training the segmentation algorithm.
Replication	The trained neural network is not fully replicable due to small changes that occur during training. Re-runs of the network training show that the network performs at the same level between different runs (<0.1% difference).
Randomization	Controls were frequency matched to the age distribution of the BBD cases in the categories of age <45, 45-55, and >55. Selection of the 13 slides for training the neural network was performed randomly, as to not bias the network.
Blinding	Investigators were blinded during data collection. Investigators were unblinded to group allocation after analyses were finalized.

Reporting for specific materials, systems and methods

We require information from authors about some types of materials, experimental systems and methods used in many studies. Here, indicate whether each material, system or method listed is relevant to your study. If you are not sure if a list item applies to your research, read the appropriate section before selecting a response.

Materials & experimental systems

n/a	Involved in the study	
☒	☐	Antibodies
☒	☐	Eukaryotic cell lines
☒	☐	Palaeontology and archaeology
☒	☐	Animals and other organisms
☒	☐	Human research participants
☒	☐	Clinical data
☒	☐	Dual use research of concern

Methods

n/a	Involved in the study	
☒	☐	ChIP-seq
☒	☐	Flow cytometry
☒	☐	MRI-based neuroimaging
