# Supplementary material for: Automated quantification of levels of breast terminal duct lobular (TDLU) involution using deep learning
Source: NPJ Breast Cancer. 2022 Jan 19;8:13. doi: 10.1038/s41523-021-00378-7 (PMC8770616; doi:10.1038/s41523-021-00378-7)
Supplement: Supplementary file 2 — Supplementary Information [file 41523_2021_378_MOESM2_ESM.pdf]

Supplementary *Note 1*

Neural Network Design:

We trained the CNN until convergence (200 epochs without improvement of validation set), occurring after 629 epochs. Epochs consisted of 100 iterations with a batch size of eight patches. Patches measuring 412 x 412 pixels were randomly sampled from the annotated regions at a resolution of 0.5 µm/pixel. The network was optimized using Adam with categorical cross-entropy loss. We set the learning rate at 0.0005 initially and reduced it by a factor of 2 each time after a loss plateau was reached for 50 epochs. We applied spatial (rotation, flipping and scaling), gaussian blurring, additive gaussian noise and color augmentations to the patches to improve the algorithm's robustness to unseen variation. Upon application, pixels were assigned the structure class with the highest probability according to the network.

Extracting quantitative features:

Before we located the predetermined TDLU locations, we performed full segmentation on the WSIs, which took around 4 minutes per slide using a setup with compute capabilities of 16 gigabytes of RAM and a NVIDIA GTX 1080 GPU.

Before extracting the measures from the segmented TDLU locations, we apply a few post-processing steps. All non-luminal pixels surrounded by the border class are transformed to the epithelium class, by performing a binary fill-hole operation (SciPy). All non-luminal pixels surrounded by the lumen class are transformed using the same operation. Afterwards, the border class is transformed to the intralobular stroma class.

The resulting TDLU segmentation is used to extract all measures. The *TDLU area* class is derived from summing the intralobular stroma, epithelium and lumen classes. The *epithelium/stroma ratio* is calculated by dividing the *epithelial area* (without *luminal area*) by the *intralobular stroma* + *epithelial area. Acini count* and *small vessel count* are calculated by performing component analysis on the corresponding segmentation classes, using the Scikit-image package available in python. For the *epithelial area*, we counted the area of the epithelium class. *Adipose tissue area and small vessel area* are calculated by taking the areas of the corresponding segmentation classes, when connected to the *TDLU area. Acini with large lumen count* was defined by a 3:1 (or higher) ratio between luminal and epithelial area of an individual lobule. The extraction of the features was carried out with Python 3.6; using the packages scikit-learn (0.20.3), NumPy (1.16.2), pandas (0.24.2), SciPy (1.2.1).

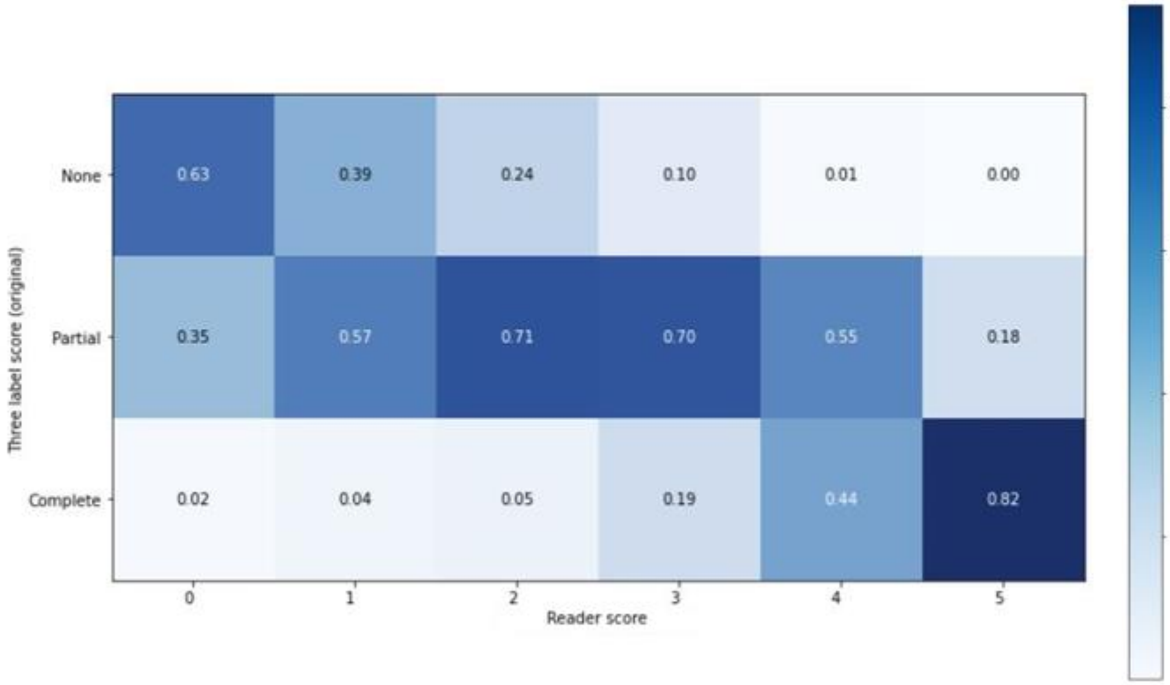

Supplementary Figure 1: Confusion matrix of the original 3-scale score (none, partial, complete) and the 6-scale score as used in the reader study of this article. The vertical axis displays the 3-scale score and the horizontal axis shows the 6-scale score. Score counts are normalized along the vertical axis.

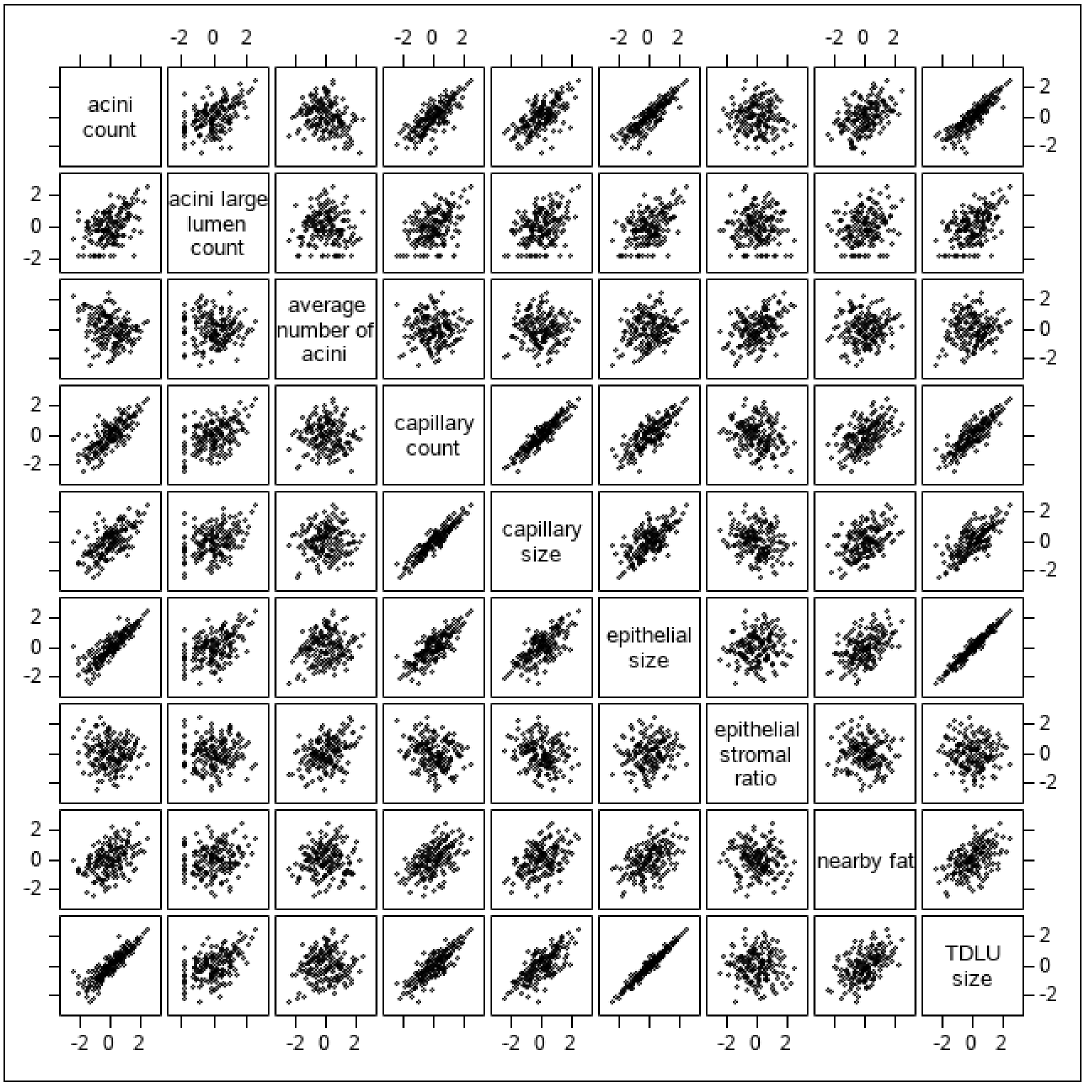

Supplementary Figure 2: Quantitative feature scatterplot: Scatterplot matrix of the quantitative features extracted by the automated method. Values were transformed using inverse normal (van der Waerden) rank scores.

Supplementary Table 1, Data collection form: The questions that were asked to the reader study participants for each individual TDLU image. The collection form consists of two sections, a tissue quality part and an involution level part.

***Quality**:*
0=Unsatisfactory, cannot be graded; 1=Satisfactory; 2=Marginal Quality, but can be graded
Concern About Partial Representation of Lobule (Secondary to Plane of Sectioning):
0=No; 1=Yes (apply to longitudinal representation of lobule; mostly duct or other features related to non-round acini in cross-section)
***No Lobule present:** tick box if applicable and put 99 in the involution level question.*
***Multiple lobules in view:** Report count if multiple lobules are fully depicted in the image. Put 99 in the involution level question if applicable.*
Involution Level:
(0) = None; Tightly packed acini, without much intervening stroma; >= 40 acini; no basement membrane thickening.
(1 or 2)= 26-39 acini; 1 vs. 2 based on packing of acini, appearance and quality of surrounding stroma. 1 if more towards normal / 2 if more towards involuted.
(3 or 4) = 10-25 acini; 3 vs. 4 based on packing of acini, appearance and quality of surrounding stroma. 3 if more towards normal / 4 if more towards involuted.
(5) = Complete Involution; <10 acini; small acini, luminal epithelium small, possibly basement thickening; surrounding stroma is densely hyalinized, vascularity inapparent; no edema.
(99)= Multiple/No lobule(s) present; Lobule cannot be graded due to quality.
· ***Acini dilated** 2-3X: tick box if applicable*
· ***Basement membrane thickening**: tick box if applicable*
· ***Acini drop out**; partial fibrous replacement: tick box if applicable*
· ***Increased inflammatory cells:** tick box if applicable*
· ***Fibrocystic Features:** tick box if applicable*

Supplementary table 2, Interobserver scores among individual readers: Interobserver scores of the four readers for the involution scores. Final column shows the kappa score of the respective

individual reader versus the consensus of the other three reviewers. Confidence intervals were obtained using bootstrapping.

	**Cohen's Kappa score (95% Confidence interval)**			
	**reader 2**	**reader 3**	**reader 4**	**Individual reviewer vs. rest**
**reader 1**	0.719 (0.718-0.720)	0.728 (0.727-0.729)	0.656 (0.655-0.657)	0.752 (0.751-0.753)
**reader 2**	x	0.725 (0.724-0.726)	0.663 (0.661-0.664)	0.749 (0.748-0.750)
**reader 3**	x	X	0.748 (0.747-0.749)	0.798 (0.797-0.799)
**reader 4**	x	X	x	0.723 (0.722-0.724)

Supplementary table 3, Associations of AI features versus demographic and clinical variables: Associations of AI-derived breast biopsy biomarker values with demographic and clinical variables.

Biomarker[1]	Attribute	Level	N	Unadjusted results		Age-adjusted results	
				Mean (SD)[2]	P-value[3]	LSMean (SE)[4]	P-value[5]
Acini count	Case status				0.071		0.019
		Case	74	0.15 (0.95)		0.18 (0.10)	
		Control	82	-0.13 (0.98)		-0.16 (0.10)	
	Lobular involution				<.001		<.001
		Complete	41	-0.85 (0.66)		-0.75 (0.12)	
		None	32	0.87 (0.89)		0.74 (0.13)	
		Partial	76	0.21 (0.68)		0.21 (0.08)	
	BBD histology				0.54		0.009
		AH	23	0.21 (0.96)		0.42 (0.18)	
		NP	64	-0.03 (1.03)		-0.24 (0.11)	

		PDWA	69	-0.04 (0.93)		0.08 (0.11)	
	High breast density				0.073		0.148
		No	46	-0.20 (0.80)		-0.15 (0.13)	
		Yes	88	0.11 (1.03)		0.08 (0.09)	
	Parous				0.819		0.392
		No	23	-0.06 (1.00)		-0.16 (0.19)	
		Yes	129	-0.01 (0.97)		0.01 (0.08)	
	Menopausal status				0.081		0.896
		Post-Menopausal	146	-0.04 (0.97)		-0.02 (0.07)	
		Pre-Menopausal	6	0.66 (0.92)		0.04 (0.38)	
	HRT Use				0.431		0.743
		Current	13	0.34 (1.20)		0.18 (0.25)	
		Ever, unsure if current	4	0.20 (0.55)		-0.15 (0.45)	
		Former	76	-0.12 (0.93)		-0.01 (0.10)	
		Never	55	-0.02 (0.97)		-0.11 (0.12)	
	ER status in cancer tissue				0.351		0.665
		Negative	15	0.39 (1.23)		0.28 (0.23)	
		Positive	48	0.12 (0.86)		0.16 (0.13)	
Acini large lumen count	Case status				0.145		0.128

		Case	74	0.12 (0.94)		0.12 (0.11)	
		Control	82	-0.11 (0.99)		-0.11 (0.11)	
	Lobular involution				0.002		0.003
		Complete	41	-0.39 (0.81)		-0.42 (0.15)	
		None	32	0.35 (1.05)		0.38 (0.18)	
		Partial	76	0.12 (0.94)		0.12 (0.11)	
	BBD histology				0.279		0.147
		AH	23	0.29 (1.08)		0.34 (0.20)	
		NP	64	-0.08 (0.94)		-0.14 (0.13)	
		PDWA	69	-0.02 (0.96)		0.01 (0.12)	
	High breast density				0.085		0.102
		No	46	-0.22 (0.89)		-0.21 (0.14)	
		Yes	88	0.08 (1.00)		0.08 (0.10)	
	Parous				0.559		0.622
		No	23	0.11 (1.04)		0.09 (0.20)	
		Yes	129	-0.02 (0.96)		-0.02 (0.09)	
	Menopausal status				0.472		0.293
		Post-Menopausal	146	0.01 (0.97)		0.01 (0.08)	
		Pre-Menopausal	6	-0.28 (0.89)		-0.44 (0.42)	
	HRT Use				0.318		0.215
		Current	13	0.23 (1.08)		0.18 (0.27)	

		Ever, unsure if current	4	-0.45 (1.35)		-0.55 (0.49)	
		Former	76	0.09 (1.00)		0.12 (0.11)	
		Never	55	-0.16 (0.89)		-0.18 (0.13)	
	ER status in cancer tissue				0.259		0.238
		Negative	15	-0.16 (0.84)		-0.18 (0.25)	
		Positive	48	0.16 (1.00)		0.17 (0.14)	
Average acinar size	Case status				0.774		0.741
		Case	74	-0.02 (0.98)		-0.03 (0.11)	
		Control	82	0.02 (0.98)		0.02 (0.11)	
	Lobular involution				0.049		0.056
		Complete	41	0.28 (1.11)		0.30 (0.16)	
		None	32	-0.29 (0.74)		-0.32 (0.18)	
		Partial	76	-0.02 (0.97)		-0.02 (0.11)	
	BBD histology				0.526		0.38
		AH	23	0.06 (0.93)		0.02 (0.21)	
		NP	64	0.09 (1.00)		0.13 (0.13)	
		PDWA	69	-0.10 (0.97)		-0.12 (0.12)	
	High breast density				0.039		0.043
		No	46	0.29 (0.83)		0.29 (0.14)	
		Yes	88	-0.07 (1.00)		-0.06 (0.10)	

	Parous				0.576		0.517
		No	23	0.08 (1.16)		0.10 (0.20)	
		Yes	129	-0.04 (0.94)		-0.05 (0.09)	
	Menopausal status				0.397		0.531
		Post-Menopausal	146	-0.01 (0.98)		-0.01 (0.08)	
		Pre-Menopausal	6	-0.35 (0.85)		-0.28 (0.42)	
	HRT Use				0.389		0.376
		Current	13	-0.37 (0.95)		-0.33 (0.27)	
		Ever, unsure if current	4	0.57 (1.16)		0.64 (0.50)	
		Former	76	-0.02 (1.01)		-0.04 (0.11)	
		Never	55	-0.03 (0.92)		-0.02 (0.13)	
	ER status in cancer tissue				0.508		0.571
		Negative	15	-0.19 (1.11)		-0.18 (0.26)	
		Positive	48	-0.00 (0.93)		-0.01 (0.14)	
Small vessel count	Case status				0.143		0.078
		Case	74	0.12 (0.92)		0.14 (0.11)	
		Control	82	-0.11 (1.02)		-0.13 (0.10)	
	Lobular involution				<.001		<.001
		Complete	41	-0.67 (0.78)		-0.61 (0.14)	
		None	32	0.66 (0.94)		0.59 (0.16)	

		Partial	76	0.15 (0.82)		0.15 (0.10)	
	BBD histology				0.139		0.009
		AH	23	0.37 (0.85)		0.52 (0.19)	
		NP	64	-0.05 (1.01)		-0.20 (0.12)	
		PDWA	69	-0.08 (0.96)		0.01 (0.11)	
	High breast density				0.247		0.402
		No	46	-0.10 (0.90)		-0.05 (0.13)	
		Yes	88	0.10 (0.97)		0.08 (0.10)	
	Parous				0.622		0.902
		No	23	0.09 (0.96)		0.02 (0.20)	
		Yes	129	-0.02 (0.97)		-0.01 (0.08)	
	Menopausal status				0.293		0.951
		Post-Menopausal	146	-0.02 (0.97)		-0.00 (0.08)	
		Pre-Menopausal	6	0.40 (0.91)		-0.03 (0.40)	
	HRT Use				0.596		0.958
		Current	13	0.03 (1.29)		-0.08 (0.26)	
		Ever, unsure if current	4	0.23 (0.65)		0.00 (0.48)	
		Former	76	-0.12 (0.95)		-0.05 (0.11)	
		Never	55	0.09 (0.93)		0.03 (0.13)	
	ER status in cancer tissue				0.897		0.644

		Negative	15	0.17 (1.27)		0.05 (0.23)	
		Positive	48	0.13 (0.87)		0.17 (0.13)	
Small vessel area	Case status				0.34		0.236
		Case	74	0.08 (0.93)		0.09 (0.11)	
		Control	82	-0.07 (1.02)		-0.09 (0.10)	
	Lobular involution				<.001		<.001
		Complete	41	-0.57 (0.84)		-0.52 (0.14)	
		None	32	0.58 (0.90)		0.52 (0.16)	
		Partial	76	0.12 (0.86)		0.11 (0.10)	
	BBD histology				0.09		0.004
		AH	23	0.41 (0.98)		0.55 (0.19)	
		NP	64	-0.09 (1.00)		-0.23 (0.12)	
		PDWA	69	-0.05 (0.93)		0.03 (0.11)	
	High breast density				0.805		0.927
		No	46	0.00 (0.95)		0.04 (0.13)	
		Yes	88	0.05 (0.95)		0.03 (0.10)	
	Parous				0.617		0.859
		No	23	0.09 (1.01)		0.03 (0.20)	
		Yes	129	-0.02 (0.97)		-0.01 (0.08)	
	Menopausal status				0.442		0.829
		Post-Menopausal	146	-0.01 (0.98)		0.00 (0.08)	

		Pre-Menopausal	6	0.30 (0.80)		-0.09 (0.41)	
	HRT Use				0.471		0.702
		Current	13	-0.16 (1.32)		-0.25 (0.27)	
		Ever, unsure if current	4	0.27 (0.56)		0.07 (0.48)	
		Former	76	-0.11 (0.94)		-0.05 (0.11)	
		Never	55	0.12 (0.94)		0.07 (0.13)	
	ER status in cancer tissue				0.891		0.506
		Negative	15	0.04 (1.21)		-0.07 (0.24)	
		Positive	48	0.08 (0.92)		0.12 (0.13)	
Epithelial area	Case status				0.169		0.066
		Case	74	0.11 (0.98)		0.14 (0.10)	
		Control	82	-0.10 (0.97)		-0.13 (0.10)	
	Lobular involution				<.001		<.001
		Complete	41	-0.72 (0.73)		-0.60 (0.13)	
		None	32	0.77 (0.83)		0.62 (0.14)	
		Partial	76	0.19 (0.79)		0.18 (0.09)	
	BBD histology				0.585		0.032
		AH	23	0.19 (1.06)		0.38 (0.19)	
		NP	64	-0.00 (0.95)		-0.20 (0.11)	
		PDWA	69	-0.06 (0.98)		0.06 (0.11)	

	High breast density				0.284		0.513
		No	46	-0.10 (0.77)		-0.04 (0.13)	
		Yes	88	0.09 (1.05)		0.06 (0.09)	
	Parous				0.727		0.848
		No	23	0.05 (1.09)		-0.05 (0.19)	
		Yes	129	-0.03 (0.95)		-0.01 (0.08)	
	Menopausal status				0.229		0.689
		Post-Menopausal	146	-0.04 (0.97)		-0.01 (0.07)	
		Pre-Menopausal	6	0.45 (0.94)		-0.17 (0.39)	
	HRT Use				0.545		0.868
		Current	13	0.21 (1.12)		0.05 (0.25)	
		Ever, unsure if current	4	0.41 (0.32)		0.07 (0.46)	
		Former	76	-0.11 (0.98)		-0.01 (0.11)	
		Never	55	-0.04 (0.93)		-0.12 (0.12)	
	ER status in cancer tissue				0.663		0.904
		Negative	15	0.23 (1.25)		0.11 (0.24)	
		Positive	48	0.10 (0.90)		0.14 (0.13)	
Epithelial stromal ratio	Case status				0.345		0.377
		Case	74	-0.08 (0.99)		-0.07 (0.11)	
		Control	82	0.07 (0.97)		0.07 (0.11)	

	Lobular involution				0.423		0.116
		Complete	41	0.15 (1.06)		0.25 (0.16)	
		None	32	-0.16 (0.82)		-0.29 (0.18)	
		Partial	76	0.00 (1.00)		-0.00 (0.11)	
	BBD histology				0.367		0.53
		AH	23	-0.19 (1.12)		-0.17 (0.21)	
		NP	64	0.12 (0.89)		0.10 (0.13)	
		PDWA	69	-0.05 (1.00)		-0.04 (0.12)	
	High breast density				0.905		0.976
		No	46	0.00 (1.09)		0.02 (0.15)	
		Yes	88	0.02 (0.93)		0.01 (0.11)	
	Parous				0.921		0.989
		No	23	0.00 (0.97)		-0.02 (0.21)	
		Yes	129	-0.02 (0.99)		-0.01 (0.09)	
	Menopausal status				0.128		0.049
		Post-Menopausal	146	0.01 (0.98)		0.02 (0.08)	
		Pre-Menopausal	6	-0.61 (0.83)		-0.83 (0.42)	
	HRT Use				0.337		0.31
		Current	13	0.23 (0.71)		0.19 (0.27)	
		Ever, unsure if current	4	0.35 (0.82)		0.27 (0.50)	

		Former	76	0.03 (0.99)		0.05 (0.12)	
		Never	55	-0.20 (1.03)		-0.22 (0.13)	
	ER status in cancer tissue				0.121		0.134
		Negative	15	0.27 (0.85)		0.26 (0.26)	
		Positive	48	-0.19 (1.02)		-0.19 (0.14)	
Adipose tissue area	Case status				0.263		0.269
		Case	74	0.09 (1.04)		0.09 (0.11)	
		Control	82	-0.08 (0.91)		-0.08 (0.11)	
	Lobular involution				0.119		0.055
		Complete	41	-0.21 (0.93)		-0.27 (0.15)	
		None	32	0.23 (0.80)		0.31 (0.18)	
		Partial	76	0.09 (0.99)		0.09 (0.11)	
	BBD histology				0.183		0.186
		AH	23	0.34 (1.08)		0.35 (0.21)	
		NP	64	-0.08 (0.93)		-0.08 (0.13)	
		PDWA	69	-0.04 (0.97)		-0.04 (0.12)	
	High breast density				0.501		0.511
		No	46	0.14 (0.84)		0.14 (0.14)	
		Yes	88	0.02 (1.04)		0.02 (0.10)	
	Parous				0.319		0.335
		No	23	-0.19 (1.19)		-0.18 (0.21)	

		Yes	129	0.04 (0.94)		0.03 (0.09)	
	Menopausal status				0.089		0.093
		Post-Menopausal	146	0.03 (0.97)		0.03 (0.08)	
		Pre-Menopausal	6	-0.67 (0.94)		-0.70 (0.42)	
	HRT Use				0.88		0.886
		Current	13	-0.09 (0.81)		-0.07 (0.27)	
		Ever, unsure if current	4	0.27 (0.86)		0.31 (0.49)	
		Former	76	0.00 (1.03)		-0.01 (0.11)	
		Never	55	-0.08 (0.93)		-0.07 (0.13)	
	ER status in cancer tissue				0.143		0.089
		Negative	15	-0.19 (1.34)		-0.25 (0.28)	
		Positive	48	0.28 (0.97)		0.30 (0.15)	
TDLU area	Case status				0.092		0.031
		Case	74	0.14 (0.94)		0.16 (0.10)	
		Control	82	-0.13 (1.00)		-0.15 (0.10)	
	Lobular involution				<.001		<.001
		Complete	41	-0.77 (0.71)		-0.67 (0.12)	
		None	32	0.79 (0.86)		0.67 (0.14)	
		Partial	76	0.20 (0.75)		0.20 (0.09)	
	BBD histology				0.475		0.016

		AH	23	0.23 (0.98)		0.42 (0.19)	
		NP	64	-0.03 (0.96)		-0.22 (0.11)	
		PDWA	69	-0.05 (0.99)		0.06 (0.11)	
	High breast density				0.314		0.547
		No	46	-0.09 (0.77)		-0.04 (0.13)	
		Yes	88	0.09 (1.04)		0.06 (0.09)	
	Parous				0.753		0.837
		No	23	0.04 (1.06)		-0.05 (0.19)	
		Yes	129	-0.03 (0.96)		-0.01 (0.08)	
	Menopausal status				0.107		0.897
		Post-Menopausal	146	-0.04 (0.97)		-0.02 (0.08)	
		Pre-Menopausal	6	0.61 (0.96)		0.04 (0.39)	
	HRT Use				0.618		0.982
		Current	13	0.13 (1.14)		-0.01 (0.25)	
		Ever, unsure if current	4	0.38 (0.34)		0.05 (0.46)	
		Former	76	-0.12 (0.99)		-0.02 (0.11)	
		Never	55	0.01 (0.92)		-0.07 (0.12)	
	ER status in cancer tissue				0.878		0.662
		Negative	15	0.19 (1.26)		0.07 (0.23)	
		Positive	48	0.15 (0.87)		0.19 (0.13)	

1. Values transformed using inverse normal (van der Waerden) rank scores. Means can be interpreted much like z-scores from a standard normal curve, in that the mean is the average number of standard deviations subjects from a given group are above the grand mean.
2. Mean and corresponding standard error
3. Unadjusted p-value
4. Age-adjusted least squares mean and corresponding standard error
5. Age-adjusted p-value

Supplementary Table 4, Breast cancer risk associations: Associations of AI-derived breast biopsy biomarkers with 5-year breast cancer risk scores.

	BBD-BC 5-year risk score (mean=3.03, SD=2.44)		Gail Model 5-year risk score (mean=2.78, SD=2.76)	
Biomarker[1]	Correlation (p-value)[2]	Partial Correlation (p-value)[3]	Correlation (p-value)[2]	Partial Correlation (p-value)[3]
Acini count	0.14 (0.071)	0.32 (<.001)	-0.13 (0.094)	0.09 (0.266)
Acini large lumen count	0.15 (0.060)	0.18 (0.021)	-0.05 (0.530)	-0.01 (0.874)
Average acini size	-0.03 (0.678)	-0.05 (0.508)	0.05 (0.575)	0.02 (0.806)
Capillary count	0.18 (0.023)	0.30 (<.001)	0.00 (0.956)	0.18 (0.028)
Capillary area	0.21 (0.009)	0.31 (<.001)	0.04 (0.644)	0.19 (0.017)
Epithelial area	0.14 (0.076)	0.30 (<.001)	-0.13 (0.107)	0.08 (0.294)
Epithelial stromal ratio	-0.05 (0.496)	-0.03 (0.700)	-0.14 (0.073)	-0.12 (0.141)
Adipose tissue area	0.22 (0.007)	0.22 (0.006)	0.15 (0.056)	0.17 (0.038)
TDLU area	0.16 (0.051)	0.31 (<.001)	-0.11 (0.176)	0.10 (0.211)

1. Values transformed using inverse normal (van der Waerden) rank scores.
2. Pearson correlation and corresponding p-value, not accounting for age.
3. Pearson correlation and corresponding p-value, partialling out the effects of age.

Supplementary Table 5, Excluded cases: Cases that were excluded from the reader study, including the case ID (anonymized) and reason for exclusion.

Case ID	Reason for exclusion
12	Used for training/development segmentation CNN

21	Used for training/development segmentation CNN
29	Used for training/development segmentation CNN
33	Used for training/development segmentation CNN
43	Error in loading Whole Slide Image file
48	Used for training/development segmentation CNN
55	Used for training/development segmentation CNN
57	Used for training/development segmentation CNN
60	Used for training/development segmentation CNN
74	Error in loading Whole Slide Image file
80	Error in loading Whole Slide Image file
101	Error in loading Whole Slide Image file
109	Used for training/development segmentation CNN
113	Used for training/development segmentation CNN
125	Used for training/development segmentation CNN
127	Used for training/development segmentation CNN
160	Used for training/development segmentation CNN
